# Gun-Related Beliefs as Predictors of Gun Policy Support: Findings from the Nationally Representative GRIP Survey

**DOI:** 10.1007/s11121-026-01883-6

**Published:** 2026-02-25

**Authors:** Julie A. Ward, Ryan Baxter-King, Phillip N. Smith, Krista R. Mehari

**Affiliations:** 1 Department of Medicine, Health, and Society, Vanderbilt College of Arts and Science, Vanderbilt University, 2301 Vanderbilt Place, Nashville, TN 37235 USA; 2 Program in Public Policy Studies, Vanderbilt College of Arts and Science, Vanderbilt University, 2301 Vanderbilt Place, Nashville, TN 37235 USA; 3 Department of Political Science, College of Liberal Arts, University of Nevada, Reno, NV USA; 4 Department of Psychology, University of South Alabama, Mobile, AL USA; 5 Department of Psychology and Human Development, Peabody College, Vanderbilt University, Nashville, TN USA

**Keywords:** Firearm policy, Public opinion, Health messaging, Public health practice, Injury prevention, Partisanship, Gun owners

## Abstract

Partisan affiliations and other demographic characteristics inadequately explain support for gun policy and provide vague public health messaging guidance. Gun-related beliefs may be more malleable and meaningful determinants of policy support for gun violence prevention. Using a nationally representative, community-engaged, mixed methods design, we examined predictive associations between gun-related beliefs and public support for six gun policies (i.e., universal background checks, waiting periods, minimum purchasing age, violent offender prohibitions, concealed carry permits, and extreme risk protection orders (ERPOs)). Gun-related beliefs were more strongly associated with policy support than political affiliation. Strength of agreement with “no one should own AR-15 style semiautomatic rifles” was positively associated with support for all six policies. Beliefs about gun policies as violence prevention were also highly salient, predicting support for five policies, with disagreement or agreement both predicting higher probability of support for universal background checks, violent offender prohibitions, and ERPOs than neutral beliefs. Beliefs that “guns are tools” were generally unassociated with policy views. Findings suggest potential for gun violence prevention messages that combine values-based language with salient themes to build support for preventive policies across political and demographic lines.

**Supplementary Information:**

The online version contains supplementary material available at 10.1007/s11121-026-01883-6.

In 2023, more than 46,000 people died from gun-related injuries in the USA (Centers for Disease Control and Prevention [CDC], 2025). Another 3–4 times as many experienced nonfatal injuries (CDC, 2025). The burden of these deaths and injuries has been unevenly borne (Kaufman et al., 2024). Young adults aged 20–34 faced the highest rates of gun death overall (CDC, 2025), and since 2020, guns have been the leading cause of death among children and adolescents (Goldstick et al., 2022). The US South experienced the highest rate of pediatric firearm injuries, with 3.5 times more emergency department visits than the region with the lowest rates of injury (Goel et al., 2023). Nationally, more than half of children hospitalized for firearm injuries in 2019 were Black youth (Goel et al., 2023). Low-income communities were also disproportionately affected (Goel et al., 2023; James et al., 2021). Higher rates of household gun ownership have been causally linked to risk for firearm suicide over time (Morral et al., 2024). However, more restrictive gun policies were associated with reductions in fatal and nonfatal injuries from suicidal, homicidal, and police-involved shootings (Schell et al., 2024; Simonetti et al., 2015; Smart et al., 2024; Ward et al., 2024).

Policy approaches to preventing gun-related deaths and injuries can include regulating gun purchases and transfers, designating lawful purchasers and possessors, promoting safer gun storage, requiring permits for public possession, designating sensitive spaces as gun-free zones, or allowing temporary firearm dispossession when indicated (Crifasi et al., 2025; Smart et al., 2024). Evidence of these policies’ effectiveness is a developing area of research (Smart et al., 2024). Still, state statutes that pair purchaser licensing systems with comprehensive background checks have been causally linked to lower violent crime rates (McCourt et al., 2020; Siegel, 2024; Smart et al., 2024). Mandatory waiting periods, another form of purchase-oriented regulation, have been shown to prevent suicides and violent crime, likely by deferring would-be impulsive acts and extending time for background check completion (Giffords Law Center, 2025; Smart et al., 2024). Raising the minimum purchasing age to 21 has been shown to lower suicide rates among youth and may prevent violent crime and mass shootings (Smart et al., 2024). Regulating public concealed carrying and prohibiting possession among people convicted of violent misdemeanors or subject to domestic violence restraining orders have each been linked to lower rates of crime and violence (Crifasi et al., 2025; Smart et al., 2024). Extreme Risk Protection Orders (ERPOs), a relatively newer policy that allows temporary dispossession and purchase prohibition if behaviors indicate extreme risk of harm to self or others, may be particularly effective for preventing suicides (Swanson et al., 2024; Zeoli et al., 2025).

Public support for stronger gun policies tends to be high (Crifasi, 2024; E. M. Stone et al., 2022). In 2023, 85% of Americans supported universal background checks, 81% supported prohibiting possession among people subject to domestic violence restraining orders, 67% supported prohibiting under-age-21 handgun possession, and 76% approved of allowing family members or healthcare providers to file ERPOs (Crifasi, 2024). Only 23% of Americans endorsed allowing people to carry a concealed firearm without a permit (Crifasi, 2024). For each of these policies, support for more restrictive conditions was lower among political Independents and Republicans than Democrats, though still represented the majority. For example. support for permitless carrying (a permissive policy) ranged from a low of 12% among Democrats to a high of 35% among Republicans (Crifasi, 2024). In addition to party affiliation, distinct patterns of gun policy support have been observed by gun ownership status (E. M. Stone et al., 2022), gender (Crifasi et al., 2021), race and ethnicity (Ward et al., 2022), age (E. M. Stone et al., 2020), and intersecting identities within (Shapira et al., 2022; Ward et al., 2022).

Research has long demonstrated that attachments to the two major US political parties can shape political attitudes, including policy support (Campbell, 1980; Green et al., 2002; Lenz, 2012; Mason, 2018; Zaller, 1992). However, partisan affiliations, gun ownership, and other social identities incompletely explain observed divergences in policy views (Burton et al., 2021) or the potentially malleable determinants of such views. In other public health issues, values-based behavioral messages have been shown to have spillover effects on policy positions, suggesting that views may be more changeable than partisan patterns may imply (Dillard et al., 2023). Beliefs about guns and gun policy, specifically, appear to develop over time, through active socialization, not limited to family exposures (Shapira et al., 2022).

Despite growing evidence and strong public favor for protective gun policies, many states lack most protective statutes (RAND, 2025). Efforts to identify demographically cross-cutting beliefs about guns and gun policies may unearth more malleable determinants of policy support, potentially enabling more effective messaging, advocacy, and coalition-building for injury prevention. To explore this potential, the objectives of this study were to (1) examine associations between gun-related beliefs and gun policy support alongside demographic identities and (2) estimate predicted support for gun policies by strength of gun-related beliefs.

## Methods

### Design

We employed an exploratory sequential (qual-->QUAN) mixed methods design, guided by principles of participatory action research to identify meaningful prevention strategies for firearm owners and other affected groups (Smith et al., 2025). These principles include directly engaging affected people and communities as collaborators throughout the research process to promote shared decision-making, mutual learning, and social change (Vaughn & Jacquez, 2020). Accordingly, this study was completed under the guidance of three advisory boards comprised of (1) gun owners, (2) residents of high-violence communities, and (3) community leaders working with violence-affected youth, all of whom resided in the Southeastern United States.

In the first phase of the study, from April 2021 to July 2023, semi-structured interviews were conducted with violence-affected and/or gun-owning residents of the US South (*n* = 250). Interviews explored participants’ gun-related beliefs, attitudes, practices, and experiences, and beliefs about injury prevention. Interviews were transcribed and analyzed to identify common themes. Drawing from these themes, survey items were developed and refined in partnership with advisory board members and community research assistants, as detailed elsewhere (Smith et al., 2025). In the second phase of the study, completed August 16, 2023, to September 29, 2023, a survey was fielded among a nationally representative sample of US adults (*n* = 1681). All survey items reflected phase-one participants’ expressed beliefs about guns and gun-related policies.

### Participants

Survey participants were recruited through an AmeriSpeak Panel via a Qualtrics contract to obtain a nationally representative sample by gender, race and ethnicity, age, and US region. The Amerispeak Panel is a nationwide probability-based sample of 97% of US households, drawn from the US Postal Service Delivery Sequence File, enhanced by targeted recruitment to under-surveyed populations. Participants in this study were AmeriSpeak Panel members, aged 18 or older, and English- or Spanish-fluent. Participants completed the survey online or by phone, incentivized by cash-equivalent AmeriSpeak points.

### Measures

Included within the survey was a module on gun-related beliefs and gun-related policy support. In this module, survey respondents were asked to rate their agreement with 8 gun-related beliefs and support for 6 gun-related policies on a 5-point Likert scale. Assessed beliefs related to general views on guns, including guns as weapons (i.e., “Guns are weapons”), guns as tools (i.e., “Guns are tools”), and guns as protection (i.e., “Guns are the best way to protect yourself”). One additional question assessed specific potential concerns about semiautomatic rifle ownership (i.e., “No one should own AR-15 style semiautomatic rifles”). Another 4 belief questions asked about general views on gun policies: as violence prevention (i.e., “Gun control laws could prevent gun violence, like mass shootings, homicides, and suicides”), as rights violations (i.e., “Gun control laws go against my Second Amendment rights”), as hurtful (i.e., “Gun control laws hurt law-abiding citizens”), or as ineffective (i.e., “Gun laws would not work because guns are already in circulation”).

Respondents also rated their support for six gun-related policies. Policy selection was informed by interview findings, with final inclusion determined by the gun owner advisory board and community research assistants’ shared views on contested issues and potential solutions. These assessed policies included universal background checks (i.e., “Requiring universal background checks for all gun sales, including those at private sales, gun shows, and pawn shops”), waiting periods (i.e., “Requiring a waiting period of 3 to 5 days before someone can buy a gun”), minimum age restrictions (i.e., “Raising the minimum age to buy any kind of gun from 18 to 21”), violent offender prohibitions (i.e., “Preventing people who have committed violent crimes from owning or using guns”), concealed carry permits (i.e., “Requiring a permit for concealed carry”), and ERPOs (i.e., “Laws that allow law enforcement officers to temporarily remove guns from people that courts say are dangerous, like those at risk for suicide”).

Respondent demographics were self-reported. Gun ownership was assessed through a single question: “Do you currently have a gun or guns?” Response options included “yes,” “no,” or “there’s a gun where I live, but it’s not mine.” “Yes” responses designated gun owners.

### Analysis

Survey weights were applied to adjust for sampling bias and non-response to generate nationally representative estimates (Table 1). Descriptive statistics were calculated to gauge respondent agreement with gun-related beliefs, policy-related beliefs, and gun policies. Next, support was dichotomized for each policy item (1 = “agree” or “strongly agree;” 0 = “neither agree nor disagree,” “disagree,” or “strongly disagree”). To identify statistically significant correlates of policy support, logistic regression models were estimated, first including only demographic covariates (Model A), then including all demographics and gun-related beliefs (Model B). “Neither agree nor disagree” was the belief referent. Finally, informed by the previously fit Model B, we calculated predicted probabilities of support for each policy by level of agreement with each gun-related belief using marginal standardization methods. This approach calculates weighted average predictions over the observed covariate distributions, rather than fixing covariates at their means. By accounting for respondent demographics and other beliefs at observed values, interpretability is improved (Muller & MacLehose, 2014).

**Table 1:** Weighted and unweighted demographic characteristics of the survey sample

	**Unweighted** ***n* (%)**	**Weighted** ***n* (%)**
**Total**	1674 (100)	1681 (100)
**Political affiliation**		
Democrat	776 (46.4)	735.8 (43.8)
Independent	288 (17.2)	300.8 (17.9)
Republican	574 (34.3)	602.8 (35.9)
**Gun owner**		
Not owner	1194 (71.3)	1166 (69.4)
Owner	466 (27.8)	505.6 (30.1)
**Gender**		
Cisgender woman	842 (50.3)	845.9 (50.3)
Cisgender man	779 (46.5)	768.1 (45.7)
Transgender woman or man, nonbinary, genderqueer, not exclusively female or male, or another gender	25 (1.5)	37.75 (2.2)
**Race and ethnicity**		
Non-Hispanic, White	1028 (61.4)	1032 (61.4)
Non-Hispanic, Black	202 (12.1)	204.5 (12.2)
Hispanic, Any Race	303 (18.1)	204.5 (17.3)
Non-Hispanic, Asian	68 (4.1)	111.4 (6.6)
Other or Multiple	73 (4.4)	42.6 (2.5)
**Age**		
18–29	299 (17.9)	334.1 (19.9)
30–44	505 (30.2)	432.4 (25.7)
45–59	373 (22.3)	392.2 (23.3)
60 +	497 (29.7)	522.4 (31.1)
**Education**		
High school diploma or less	404 (24.1)	629.7 (37.5)
Some college	626 (37.4)	447.5 (26.6)
Bachelor’s degree or higher	644 (38.5)	603.9 (35.9)
**Marital Status**		
Married	805 (48.1)	842.4 (50.1)
Never married	569 (34.0)	537.4 (32.0)
Other	300 (17.9)	301.2 (17.9)
**Income**		
Less than $30,000	366 (21.9)	351.7 (20.9)
$30,000 to under $60,000	425 (25.4)	434.6 (25.9)
$60,000 to under $100,000	416 (24.9)	414.6 (24.7)
$100,000 or more	467 (27.9)	480.2 (28.6)
**Current employment status**		
Employed	1075 (64.2)	1065 (63.4)
Unemployed, looking for work	71 (4.2)	76.2 (4.5)
Not working, retired	284 (17.0)	314.4 (18.7)
Not working, other (includes disabled, temporary layoff)	244 (14.6)	225.2 (13.4)
**Region**		
Northeast	207 (12.4)	289 (17.2)
Midwest	421 (25.8)	347.5 (20.7)
South	562 (33.6)	647.6 (38.5)
West	474 (28.3)	397 (23.6)
**Urbanicity**		
Nonmetropolitan area	223 (13.3)	236.3 (14.1)
Metropolitan area	1451 (86.7)	1445 (85.9)

Results are presented with 95% confidence intervals and statistical significance defined by *p* <.05. Analyses were conducted using the *svy* and *margins* commands in Stata, version 16.1. The study was reviewed and approved by the IRBs of the University of South Alabama prior to data collection and Vanderbilt University for secondary data analysis.

## Results

### Strength of Agreement

Most respondents agreed or strongly agreed with the assessed gun policies. The policy with the smallest majority agreement involved minimum age restrictions (agree = 22%, strongly agree = 43%). Policies with the highest agreement were violent offender prohibitions (agree = 27%, strongly agree = 55%) and universal background checks (agree = 27%, strongly agree = 55%). Agreement and strong agreement with concealed carry permits, waiting periods, and ERPOs were comparable. For all assessed policies, 43–55% of respondents reported strong agreement; 65–82% reported agreement or strong agreement (Table 2).

**Table 2:** Strength of agreement with gun-related belief and policy items (weighted)

	**Strongly disagree** ***%* (95% CI)**	**Disagree** ***%* (95% CI)**	**Neither agree nor disagree** ***%* (95% CI)**	**Agree** ***%* (95% CI)**	**Strongly agree** ***%* (95% CI)**
**Gun-related beliefs**					
Guns are weapons.^a^	1.3 (0.8, 2.1)	1.4 (0.9, 2.3)	10.9 (8.9, 13.2)	47.3 (44.1, 50.5)	38.7 (35.7, 41.9)
Guns are tools.^b^	6.6 (5.1, 8.5)	13.5 (11.4, 15.6)	22.2 (19.6, 25.1)	39.0 (36.0, 42.1)	18.5 (16.1, 21.1)
Guns are the best way to protect yourself.^c^	11.5 (9.6, 13.6)	20.9 (18.5, 24.4)	33.4 (30.4, 36.6)	21.4 (18.9, 24.2)	12.8 (10.8, 15.1)
No one should own AR-15-style semiautomatic rifles.^d^	12.6 (10.7, 14.7)	11.2 (9.2, 13.5)	21.4 (18.8, 24.2)	17.0 (14.7, 19.4)	37.5 (34.4, 40.6)
**Policy-related beliefs**					
Gun control laws could prevent gun violence, like mass shootings, homicides, and suicides.^e^	16.4 (14.0, 19.1)	14.0 (12.0, 16.4)	21.7 (19.1, 24.5)	24.9 (22.4, 27.6)	22.6 (20.0, 25.4)
Gun control laws go against my Second Amendment rights.^f^	21.3 (18.8, 24.0)	19.1 (16.8, 21.6)	32.4 (29.4, 35.5)	15.2 (13.1, 17.6)	11.7 (9.7, 14.1)
Gun control laws hurt law-abiding citizens.^g^	21.1 (18.6, 23.9)	19.7 (17.3, 22.2)	26.5 (23.8, 29.5)	19.0 (16.6, 21.7)	13.3 (11.2, 15.6)
Gun laws would not work because guns are already in circulation.^h^	14.4 (12.3, 16.7)	19.4 (17.1, 22.0)	31.3 (28.3, 34.4)	23.7 (21.1, 26.5)	10.8 (9.0, 12.9)
**Gun-related policies**					
Requiring universal background checks for all gun sales, including those at private sales, gun shows, and pawn shops.^i^	2.8 (1.8, 4.1)	2.9 (2.1, 4.1)	11.9 (10.0, 14.2)	26.7 (23.9, 29.8)	55.0 (51.8, 58.2)
Requiring a waiting period of 3 to 5 days before someone can buy a gun.^j^	4.0 (2.9, 5.6)	5.3 (4.1, 7.0)	19.1 (16.6, 21.9)	22.5 (19.9, 25.3)	48.4 (45.3, 51.6)
Raising the minimum age to buy any kind of gun from 18 to 21.^k^	5.9 (4.5, 7.5)	6.9 (5.3, 8.8)	21.6 (19.0, 24.4)	22.3 (19.7, 25.1)	43.0 (39.9, 46.2)
Preventing people who have committed violent crimes from owning or using guns.^l^	1.8 (1.1, 3.1)	2.0 (1.2, 3.1)	14.2 (12.1, 16.7)	26.8 (24.0, 29.7)	54.7 (51.4, 57.8)
Requiring a permit for concealed carry.^m^	4.5 (3.3, 6.1)	4.4 (3.2, 5.8)	15.2 (12.9, 17.8)	25.1 (22.4, 28.0)	50.2 (47.0, 53.4)
Laws that allow law enforcement officers to temporarily remove guns from people that courts say are dangerous, like those at risk for suicide.^n^	3.7 (2.7, 5.1)	3.8 (2.9, 5.0)	20.1 (17.7, 22.8)	28.1 (25.2, 31.2)	43.7 (40.6, 46.9)

^a^In the narrative, this item is described as “guns as weapons.” ^b^In the narrative, this item is described as “guns as tools.” ^c^In the narrative, this item is described as “guns as protection.” ^d^In the narrative, this item is described as “concerns about semiautomatic rifle ownership.” ^e^In the narrative, this item is described as “gun policies as violence prevention.” ^f^In the narrative, this item is described as “gun policies as rights violations.” ^g^In the narrative, this item is described as “gun policies as hurtful.” ^h^In the narrative, this item is described as “gun policies as ineffective.” ^i^In the narrative, this item is described as “universal background checks.” ^j^In the narrative, this item is described as “waiting periods.” ^k^In the narrative, this item is described as “minimum age restrictions.” ^l^In the narrative, this item is described as “violent offender prohibitions.” ^m^In the narrative, this item is described as “concealed carry permits.” ^n^In the narrative, this item is described as “extreme risk protection orders (ERPOs).”

Strength of agreement with gun-related beliefs was more varied. For two assessed beliefs (i.e., guns as protection and gun policies as ineffective), responses followed a normal distribution with 33% and 31%, respectively, neither agreeing nor disagreeing with these statements. In response to these items, similar proportions of respondents agreed or disagreed and strongly agreed or strongly disagreed. For two other beliefs—regarding gun policies as rights violations and gun policies as hurtful—similar portions of respondents were neutral. However, more respondents disagreed (19% and 20%, respectively) or strongly disagreed (21% for each) than agreed or strongly agreed. For the four remaining items, fewer respondents expressed neutral views. Instead, respondents largely agreed or strongly agreed with these beliefs (i.e., guns as tools, agree = 39%, strongly agree = 19%; guns as weapons, agree = 47%, strongly agree: 39%; gun policies as violence prevention, agree = 25%, strongly agree = 23%; concerns about semiautomatic rifle ownership, agree = 17%, strongly agree = 38%) (Table 2).

**Fig. 1:**
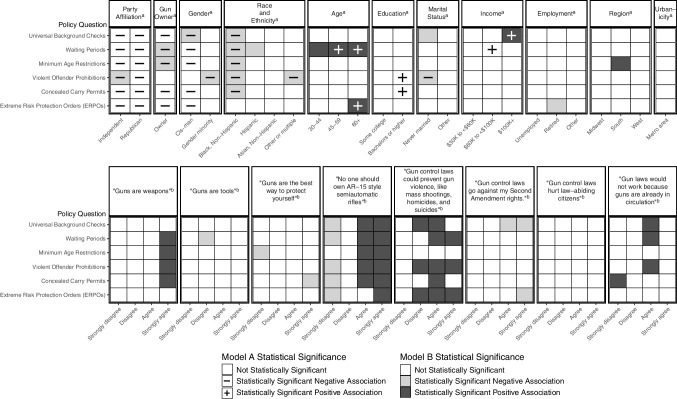
Demographic- and belief-related correlates of gun policy support in logistic regression models. Note: Symbols denote statistically significant association with policy support in demographics-only model (Model A); shading denotes statistically significant association with policy support in the demographics and beliefs model (Model B). Statistical significance was defined as *p* ≤ 0.05. ^a^For party identity, the referent was Democrat; for gun ownership, the referent was non-owner; for race and ethnicity, the referent was White, non-Hispanic; for age, the referent was ages 18–29; for education, the referent was high school or less; for income, the referent was less than $30 K; for employment, the referent was employed; for region, the referent was Northeast; for urbanicity, the referent was non-metro area. ^b^For all beliefs, the referent was “neither agree nor disagree.”

Multiple demographic characteristics and gun-related beliefs were associated with belief-and-policy paired responses. Race and ethnicity, age, marital status, and gun ownership were among the most significant. Gun ownership, party affiliation, and gender were less frequently significantly associated with policy support when gun-related beliefs were included as covariates (Fig. 1; Supplemental Tables 1 and 2). Predicted probability models included all statistically significant and non-significant demographics and beliefs.

### Predicted Probabilities

**Fig. 2:**
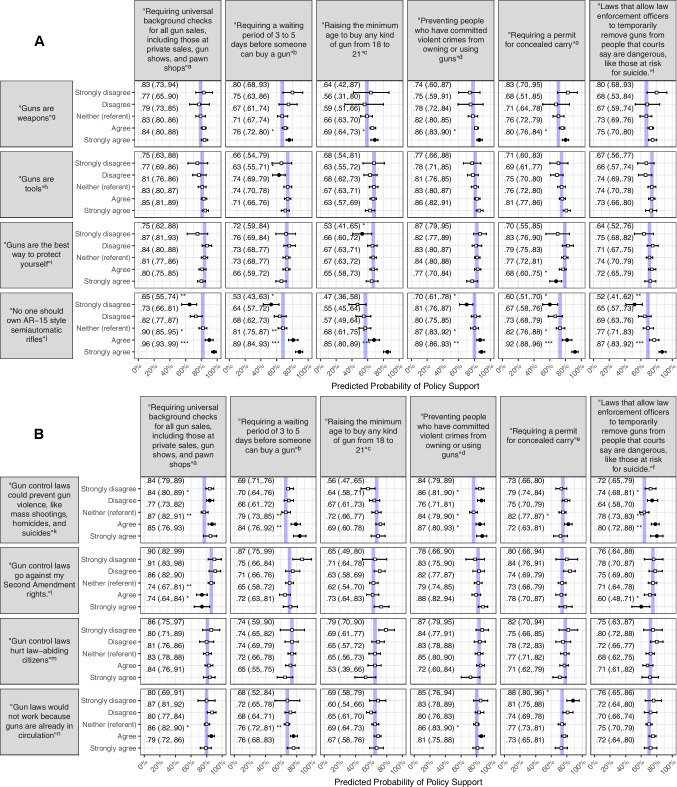
Predicted probability of support for each gun-related policy, **A** given strength of agreement with each gun-related belief; **B** given strength of agreement with each policy-related belief (weighted). Note:*p ≤ 0.05,**p ≤ 0.01,***p ≤ 0.001 **Bold marker shading indicates statistical significance** (*p* ≤ 0.05)**.** Neither agree nor disagree is the referent, value indicated by the vertical line. Predicted probability models hold all demographic characteristics (i.e., respondent age, gender, race and ethnicity, education, employment, marital status, income, US region, urbanicity, political party affiliation, gun ownership status) and respondent strength of agreement with other gun-related and policy-related beliefs at observed values. ^a^In the narrative, this item is described as universal background checks. ^b^In the narrative, this item is described as waiting periods. ^c^In the narrative, this item is described as minimum age restrictions. ^d^In the narrative, this item is described as violent offender prohibitions. ^e^In the narrative, this item is described as concealed carry permits. ^f^In the narrative, this item is described as extreme risk protection orders (ERPOs). ^g^In the narrative, this item is described as “guns as weapons.” ^h^In the narrative, this item is described as “guns as tools.” ^i^In the narrative, this item is described as “guns as protection.” ^j^In the narrative, this item is described as “concerns about semiautomatic rifle ownership.” ^k^In the narrative, this item is described as “gun policies as violence prevention.” ^l^In the narrative, this item is described as “gun policies as rights violations.” ^m^In the narrative, this item is described as “gun policies as hurtful.” ^n^In the narrative, this item is described as “gun policies as ineffective.”

After accounting for observed demographic characteristics and gun-related beliefs, gun policy support was most consistently and strongly predicted by views about semiautomatic rifles. For example, agreement with the statement “no one should own AR-15 style semiautomatic rifles” predicted 68% probability of support for minimum age policies (95% CI = 0.61–0.75); strong agreement predicted 85% probability of support (95% CI = 0.80–0.89). For ERPO policies, strong agreement with such concerns predicted 87% probability of support (95% CI = 0.83–0.92), whereas strong disagreement predicted 52% probability of support (95% CI = 0.41–0.62). For all other policies, strong disagreement with semiautomatic rifle concerns predicted statistically significantly lower support, and agreement or strong agreement predicted significantly higher probability of support, relative to neutral beliefs. Predicted probability of support was 92% for concealed carry permits in association with strong agreement (95% CI = 0.88–0.96) and 96% for universal background checks (strong agreement 95% CI = 0.93–0.99) (Fig. 2A).

Among other gun-related beliefs, strongly agreeing that “guns are weapons” was associated with a significantly higher probability of support for waiting periods (76%, 95% CI = 0.72–0.80), minimum age restrictions (69%, 95% CI = 0.64–0.73), violent offender prohibitions (86%, 95% CI = 0.83–0.90), and concealed carry permits (80%, 95% CI = 0.76–0.84). Beliefs about guns as tools, regardless of strength or direction, were largely not significantly associated with predicted policy support. One exception was for waiting periods, with disagreement predicting somewhat lower support (63%, 95% CI = 0.55–0.71). Beliefs about guns as protection also rarely predicted policy views, except in two extreme cases: relative to neutral beliefs, strong disagreement predicted lower probability of support for minimum age requirements (53%, 95% CI = 0.41–0.65), and strong agreement predicted lower probability of support for concealed carry permits (68%, 95% CI = 0.60–0.75) (Fig. 2A).

Among policy-related beliefs, gun policies as violence prevention predicted support for all assessed policies except minimum age restrictions. Notably, in association with this belief, a striking pattern emerged to reveal a curvilinear association with three policies. Specifically, for universal background checks, violent offender prohibitions, and ERPOs, both disagreement and agreement predicted higher probability of support, relative to neutral beliefs. For example, neutral beliefs about gun policies as violence prevention predicted 64% probability of ERPO support, the lowest of all belief positions. In contrast, disagreement predicted 74% probability (95% CI = 0.68–0.81), agreement predicted 78% probability (95% CI = 0.73–0.83), and strong agreement predicted 80% probability of support (95% CI = 0.72–0.88). Support for violent offender prohibitions followed a similar pattern, with neutral views predicting 76% probability of support (95% CI = 0.71–0.81) and disagreement predicting higher probability of support (86%, 95% CI = 0.81–0.90), as did agreement (84%, 95% CI = 0.79–0.90) or strong agreement (87%, 95% CI = 0.80–0.93). The pattern for universal background checks was similar (Fig. 2B).

Agreeing or strongly agreeing with beliefs about gun policies as rights violations predicted significantly lower probability of support for background checks (agree = 74%, 95% CI = 0.67–0.81; strongly agree = 74%, 95% CI = 0.64–0.84). Strong agreement also predicted lower support for ERPOs (60%, 95% CI = 0.48–0.71). Strongly disagreeing with gun policies as ineffective predicted high probability of support for concealed carry permits (88%, 95% CI = 0.80–0.96). Somewhat paradoxically, agreement also predicted higher probability of support for background checks (86%, 95% CI = 0.82–0.90), waiting periods (76%, 95% CI = 0.72–0.81), and violent offender prohibitions (86%, 95% CI = 0.83–0.90). Beliefs about gun policies as harmful to law-abiding citizens were not significantly associated with predicted policy support (Fig. 2B).

## Discussion

In this analysis of the relationship between gun-related beliefs and support for gun policy, we identified two particularly salient beliefs. First, strength of agreement with the belief that “No one should own AR-15 style semiautomatic rifles” positively predicted support for universal background checks, waiting periods, minimum age requirements, violent offender prohibitions, concealed carry permits, and ERPO statutes. After accounting for other beliefs and respondent demographics, associations were nearly linear, with strong disagreement predicting lower probability of support (e.g., 53% for waiting periods) and agreement or strong agreement predicting higher probability of support (e.g., 81% and 89% for waiting periods, respectively). Research by Rogowski and Tucker (2019) suggests that emerging adults’ views about gun policies develop, in part, through conversations about gun violence in the wake of high-profile shootings. Semiautomatic rifle use is rare in gun homicides overall, but is disproportionately involved in mass-casualty events (E. Stone et al., 2024). The strong association between concerns about semiautomatic rifle ownership and predicted support for all six assessed gun policies suggests that messaging tailored to beliefs about semiautomatic rifles may have resonance that extends beyond event-specific discourse or policies that directly target assault weapons. This finding may inform efforts to develop public health messages that inspire broader support for collective prevention, as seen in studies of pandemic-era prosocial emotional appeals (Heffner et al., 2021).

Strength of agreement with the belief that “gun control laws could prevent gun violence like mass shootings, homicides, and suicides” was also notably predictive of support for five of six gun policies. However, this belief tended to display a more curvilinear association, with disagreement and agreement both suggesting a higher probability of support for universal background checks, violent offender prohibitions, and ERPOs than respondents with neutral views. This seemingly paradoxical finding is consistent with prior research suggesting that advocacy intentions and behaviors may be strengthened at both high and low levels of attitude certainty (Cheatham & Tormala, 2017). In other words, the more certain an individual is about an attitude, in this case regarding policies’ preventive potential, the more likely they are to act accordingly; as beliefs shift to lower certainty, or potentially from strongly disagree to disagree, behavioral intentions may be renewed by information-seeking motivations (Cheatham & Tormala, 2017). In the context of gun policy, people who strongly endorsed gun policies as violence prevention had 80% probability of supporting ERPOs and 87% probability of supporting violent offender prohibitions. Probability of support was more than ten points lower for neutral responders. People who disagreed, but not strongly disagreed, again showed a higher probability of support (ERPOs = 74%, violent offender prohibitions = 86%).

The identified link between policy support and beliefs about gun policy as violence prevention may be variously interpreted. First, the curvilinear association may suggest that public education about gun policy research could promote information-seeking in ways that are both directly and indirectly associated with support. Alternatively, responses may reveal critical insight into the public’s interpretation of “prevention” as unrealistic, implying elimination rather than reduction. In spring 2024, the CDC held focus groups to test the effect of new gun-related public health messages. Among them was a message stating “We can prevent gun injury and death. It’s not inevitable.” In response to this message, participants reported skepticism about whether prevention, which many interpreted as eradication, was possible. The more popular message reframed prevention to reduction, focusing on making gun injuries “less likely” through proven solutions (Dills et al., 2024). Knowledge-based interventions alone are typically insufficient for achieving desired behaviors (Arlinghaus & Johnston, 2018; Khullar & Young, 2025). Even so, by either explanation, improving awareness of effective prevention strategies through educational campaigns may prove beneficial for achieving broader or more activated policy support, even if some uncertainties remain (Mantzari et al., 2022; Nagler et al., 2023).

Other assessed beliefs showed only occasionally significant predictive associations with policy support—typically at the extremes. For example, after accounting for other beliefs and demographics, strongly agreeing with “guns are weapons” was significantly associated with higher predicted support for waiting periods, minimum purchasing age, violent offender prohibitions, and concealed carry permits. Other positions were not statistically distinguishable from neutral. Conversely, the belief that “guns are the best way to protect yourself” predicted significantly lower support for minimum age requirements or concealed carry permits among people who expressed strong disagreement or strong agreement, respectively. Agreement with “guns are tools,” a common framing among gun owners (Shapira & Simon, 2018), was generally unassociated with policy positions. Moreover, concerns about rights infringements were not significantly associated with most policies, and potential harms to law-abiding citizens showed no association with predicted policy endorsement. Collectively, these findings suggest potential for using values-based language to connect with gun-owning audiences (Khullar & Young, 2025) without necessarily shaping policy-specific views. Future research should test the effect of combining such framing with more salient themes on message acceptance and policy support.

A call for more effective, information-based campaigns may be challenged by multiple coinciding national trends. First, despite generally high expressed support for most gun policies (Crifasi, 2024), the national trend has been toward policy relaxation (Siegel et al., 2017) or stagnation, facilitated by preemption laws to concentrate gun regulation at the state, not local, level (Pomeranz & Ochoa, 2021). Continuing to raise awareness about the public’s support for protective gun-related policies, as indicated by this study and others, may politically empower state legislators to advance these policies. Another challenge is growing disagreement about facts, alongside pervasive reliance on opinions and personal experience, which has led to greater uncertainty, loss of civil discourse, lower institutional trust, and political stalemates (Kavanagh & Rich, 2018). The relatively large portion of “neither agree nor disagree” responses to gun-related beliefs may be indicative of these coinciding trends in the gun policy realm. Such conditions may deepen reliance on information-sharing through trusted relationships and expert opinion for successful public health messaging, mobilization, and prevention. Research suggests that many patients are open to conversations with healthcare providers about gun safety (Pallin et al., 2019). However, opportunity remains to strengthen clinicians’ confidence and competence in discussing individual or collective firearm injury prevention strategies (Conner et al., 2021; Sykes et al., 2023).

The results presented should be considered in the context of some limitations. First, although prior research suggests a plausible link between beliefs and policy views, a causal relationship cannot be inferred from this cross-sectional design. Future research should test the effect of belief-based messaging on gun policy support and advocacy intentions. Second, community members were engaged throughout the research process to protect against perceived bias and maintain a focus on issues of importance to affected groups. Still, some phrasing, such as “gun laws would not work because guns are already in circulation,” may have been unintentionally confusing or double-barreled. Alternative phrasing may yield different results. Finally, the risk for sampling bias and survey non-response is ever-present in survey designs. The AmeriSpeak panel’s probability-based sampling frame and applied survey-based weights mitigated this risk. Evidence of strong alignment between this study’s assessed policy support and prior national estimates provides further assurance of both internal and external validity.

This study used a rigorous mixed methods design to test agreement with gun-related beliefs and gun policy support, informed by the interests and concerns of gun-owning and violence-affected residents. Results suggest that several gun-related beliefs predict favor for gun policy, over and above historically assessed demographic correlates of policy support. These associations suggest potential for health and policy advocates, as well as policymakers themselves, to leverage evidence-informed, values-based messages to build further coalitional support to advance protective gun policies. For example, messages that activate beliefs about semiautomatic rifles or provoke opinions on policy as violence prevention may increase receptivity to specific evidence-informed gun policies. It is also possible that even polarizing topics, such as second amendment rights, could be neutrally acknowledged when introducing specific policy or legislation, such as violent offender prohibitions or waiting periods, without directly shaping policy positions (e.g., “We need to protect second amendment rights by ensuring that only responsible citizens who have the right to own firearms are purchasing firearms.”). However, further research is needed to test the impact of activating beliefs on individuals’ support for particular policies, beginning with participatory research to develop messaging, focus groups to qualitatively explore how those messages are experienced, and experiments to test the impact of messaging on individuals’ support for specific policies. Future research should build on these findings to test the malleability of respondents’ gun-related beliefs and the effect of targeted messages on activated support for evidence-informed gun policy.

## Supplementary Information

Below is the link to the electronic supplementary material.

ESM 1 (PDF 294 KB)

## Data Availability

Data can be made available upon reasonable request submitted to krista.mehari@vanderbilt.edu.
