# Supplementary material for: Gun-Related Beliefs as Predictors of Gun Policy Support: Findings from the Nationally Representative GRIP Survey
Source: Prev Sci. 2026 Feb 25;27(2):347–58. doi: 10.1007/s11121-026-01883-6 (PMC12999767; doi:10.1007/s11121-026-01883-6)
Supplement: Supplementary file 1 — (PDF 294 KB) [file 11121_2026_1883_MOESM1_ESM.pdf]

Gun-related beliefs as predictors of gun policy support: Findings from the nationally representative GRIP survey

Julie A. Ward, Ryan Baxter-King, Phillip N. Smith, Krista R. Mehari

Table of Contents

Supplemental Table 1. **..1**

Supplemental Table 2. **..4**

Supplemental Table 1. Odds of gun policy support, adjusted for respondent demographics only (Model A) (weighted, *n*=1602)

	Universal background checks[a]	Waiting periods[b]	Minimum age restrictions[c]	Violent offender prohibitions[d]	Concealed carry permits[e]	Extreme Risk Protection Orders (ERPOs)[f]
**Political Affiliation**						
Democrat	*Ref*	*Ref*	*Ref*	*Ref*	*Ref*	*Ref*
Independent	0.24*** (0.14, 0.42)	0.28*** (0.18, 0.46)	0.34*** (0.22, 0.53)	0.27*** (0.16, 0.45)	0.27*** (0.17, 0.44)	0.27*** (0.17, 0.43)
Republican	0.18*** (0.11, 0.29)	0.19*** (0.13, 0.28)	0.25*** (0.17, 0.35)	0.40*** (0.24, 0.67)	0.20*** (0.13, 0.30)	0.22*** (0.15, 0.32)
**Gun Ownership Status**						
Non-owner	*Ref*	*Ref*	*Ref*	*Ref*	*Ref*	*Ref*
Owner	0.56* (0.35, 0.90)	0.28*** (0.19, 0.41)	0.38*** (0.27, 0.53)	0.75 (0.48, 1.17)	0.41*** (0.27, 0.61)	0.55*** (0.38, 0.79)
**Gender**						
Cis-woman	*Ref*	*Ref*	*Ref*	*Ref*	*Ref*	*Ref*
Cis-man	0.40*** (0.26, 0.59)	0.54*** (0.38, 0.76)	0.59*** (0.44, 0.81)	0.81 (0.54, 1.20)	0.49*** (0.35, 0.70)	0.60** (0.43, 0.85)
Gender minority[g]	0.50 (0.07, 3.63)	0.41 (0.12, 1.30)	0.66 (0.20, 2.22)	0.19** (0.06, 0.60)	0.97 (0.12, 7.56)	1.04 (0.31, 3.51)
**Race and Ethnicity**						
White, non-Hispanic	*Ref*	*Ref*	*Ref*	*Ref*	*Ref*	*Ref*
Black, non-Hispanic	0.42* (0.21, 0.83)	0.42** (0.23, 0.75)	0.52* (0.31, 0.90)	0.13*** (0.07, 0.22)	0.41*** (0.24, 0.70)	0.63 (0.35, 1.15)
Hispanic	0.74 (0.41, 1.35)	0.64 (0.39, 1.04)	1.34 (0.84, 2.15)	0.63 (0.37, 1.08)	0.82 (0.48, 1.41)	1.31 (0.82, 2.10)
Asian, non-Hispanic	0.97 (0.39, 2.40)	0.77 (0.34, 1.78)	1.41 (0.64, 3.10)	1.53 (0.56, 4.18)	0.99 (0.37, 2.64)	1.89 (0.75, 4.77)
Other or multiple	0.94 (0.42, 2.13)	0.90 (0.46, 1.76)	1.15 (0.57, 2.34)	0.31** (0.14, 0.67)	0.83 (0.38, 1.81)	1.05 (0.52, 2.11)
**Age**						
18-29	*Ref*	*Ref*	*Ref*	*Ref*	*Ref*	*Ref*
30-44	0.94 (0.49, 1.83)	1.72 (0.98, 2.99)	0.89 (0.53, 1.48)	0.67 (0.39, 1.15)	0.67 (0.38, 1.20)	1.05 (0.63, 1.75)
45-59	1.37 (0.67, 2.80)	2.06* (1.13, 3.76)	0.89 (0.52, 1.50)	1.01 (0.55, 1.85)	0.98 (0.53, 1.78)	1.25 (0.71, 2.20)
60+	1.24 (0.54, 2.83)	3.49*** (1.66, 7.34)	0.98 (0.54, 1.78)	1.41 (0.58, 3.40)	1.45 (0.69, 3.08)	2.61** (1.31, 5.21)

**Education**						
High school or less	*Ref*	*Ref*	*Ref*	*Ref*	*Ref*	*Ref*
Some college	0.99 (0.63, 1.56)	1.22 (0.82, 1.82)	1.28 (0.88, 1.86)	1.01 (0.65, 1.58)	1.05 (0.70, 1.59)	0.78 (0.53, 1.15)
Bachelor's degree or higher	1.45 (0.81, 2.60)	1.31 (0.81, 2.12)	1.47 (0.96, 2.27)	2.00* (1.16, 3.47)	2.09** (1.27, 3.44)	1.54 (0.96, 2.49)
**Marital Status**						
Married	*Ref*	*Ref*	*Ref*	*Ref*	*Ref*	*Ref*
Never married	0.65 (0.38, 1.10)	0.94 (0.58, 1.50)	0.72 (0.47, 1.09)	0.54* (0.34, 0.87)	0.88 (0.55, 1.40)	0.78 (0.50, 1.22)
Other	1.03 (0.59, 1.79)	1.08 (0.69, 1.71)	1.07 (0.68, 1.69)	1.12 (0.62, 2.05)	1.02 (0.62, 1.67)	1.07 (0.65, 1.75)
**Income**						
Under $30,000	*Ref*	*Ref*	*Ref*	*Ref*	*Ref*	*Ref*
$30,000 to under $60,000	0.99 (0.58, 1.70)	1.24 (0.78, 1.98)	0.77 (0.49, 1.23)	1.07 (0.64, 1.78)	1.33 (0.79, 2.22)	1.10 (0.70, 1.75)
$60,000 to under $100,000	1.01 (0.55, 1.85)	1.76* (1.06, 2.93)	0.76 (0.48, 1.23)	0.93 (0.54, 1.61)	1.11 (0.65, 1.92)	1.05 (0.62, 1.80)
$100,000 or more	2.19* (1.11, 4.29)	1.67 (0.97, 2.85)	1.03 (0.62, 1.72)	1.35 (0.74, 2.46)	1.69 (0.94, 3.02)	1.26 (0.73, 2.17)
**Employment**						
Employed	*Ref*	*Ref*	*Ref*	*Ref*	*Ref*	*Ref*
Unemployed, looking for work	0.79 (0.34, 1.83)	1.40 (0.62, 3.17)	0.99 (0.49, 1.99)	1.14 (0.50, 2.59)	0.72 (0.33, 1.57)	1.28 (0.58, 2.84)
Retired	1.70 (0.83, 3.49)	1.34 (0.73, 2.46)	1.29 (0.75, 2.22)	1.64 (0.72, 3.74)	1.10 (0.57, 2.14)	0.60 (0.33, 1.08)
Other	0.79 (0.44, 1.40)	0.81 (0.51, 1.30)	0.87 (0.56, 1.37)	0.81 (0.48, 1.37)	0.76 (0.45, 1.27)	0.78 (0.49, 1.25)
**Region**						
Northeast	*Ref*	*Ref*	*Ref*	*Ref*	*Ref*	*Ref*
Midwest	0.65 (0.33, 1.30)	0.90 (0.53, 1.54)	0.84 (0.52, 1.37)	0.72 (0.37, 1.37)	0.63 (0.34, 1.18)	0.66 (0.37, 1.18)
South	0.76 (0.38, 1.52)	1.18 (0.71, 1.96)	1.53 (0.95, 2.48)	0.88 (0.48, 1.61)	0.98 (0.52, 1.83)	0.78 (0.45, 1.35)
West	0.59 (0.29, 1.18)	1.08 (0.62, 1.86)	1.05 (0.64, 1.71)	0.95 (0.50, 1.79)	0.74 (0.38, 1.47)	0.60 (0.35, 1.04)
**Urbanicity**						

Non-metro area	*Ref*	*Ref*	*Ref*	*Ref*	*Ref*	*Ref*
Metro Area	1.05 (0.64, 1.71)	0.99 (0.63, 1.57)	1.44 (0.96, 2.17)	0.93 (0.54, 1.60)	1.15 (0.73, 1.81)	1.29 (0.84, 1.99)

Note:*= p≤0.05,**= p≤0.01,***= p≤0.001

a = “Requiring universal background checks for all gun sales, including those at private sales, gun shows, and pawn shops.” b = “Requiring a waiting period of 3 to 5 days before someone can buy a gun.” c = “Raising the minimum age to buy any kind of gun from 18 to 21.” d = “Preventing people who have committed violent crimes from owning or using guns.” e = “Requiring a permit for concealed carry.” f = “Laws that allow law enforcement officers to temporarily remove guns from people that courts say are dangerous, like those at risk for suicide.” g = “Gender minority” represents respondents who self-identified as transmasculine, transfeminine, nonbinary, genderqueer, not exclusively female or male, or another gender.

Supplemental Table 2. Odds of gun policy support, adjusted for respondent demographics and beliefs (Model B) (weighted, *n*=1602)

	Universal background checks[a]	Waiting periods[b]	Minimum age restrictions[c]	Violent offender prohibitions[d]	Concealed carry permits[e]	Extreme Risk Protection Orders (ERPOs)[f]
**"Guns are weapons"**						
Strongly disagree	1.52 (0.45, 5.16)	3.06 (0.81, 11.53)	1.41 (0.32, 6.22)	0.69 (0.21, 2.26)	2.61 (0.69, 9.89)	2.76 (0.84, 9.08)
Disagree	0.84 (0.24, 2.97)	1.80 (0.58, 5.58)	0.84 (0.19, 3.81)	0.78 (0.20, 3.01)	0.77 (0.20, 2.94)	1.13 (0.36, 3.55)
Neither agree nor disagree	*Ref*	*Ref*	*Ref*	*Ref*	*Ref*	*Ref*
Agree	1.45 (0.76, 2.76)	1.29 (0.72, 2.30)	1.60 (0.96, 2.66)	1.43 (0.78, 2.62)	1.41 (0.79, 2.52)	1.49 (0.86, 2.56)
Strongly agree	1.62 (0.80, 3.30)	2.03* (1.04, 3.94)	1.86* (1.06, 3.27)	2.24* (1.14, 4.42)	2.02* (1.06, 3.87)	1.82 (0.99, 3.32)
**"Guns are tools"**						
Strongly disagree	0.58 (0.17, 1.94)	0.53 (0.18, 1.60)	0.99 (0.39, 2.51)	0.73 (0.26, 2.02)	0.71 (0.26, 1.99)	0.63 (0.28, 1.42)
Disagree	0.69 (0.27, 1.74)	0.40* (0.19, 0.85)	0.74 (0.39, 1.42)	0.80 (0.38, 1.71)	0.61 (0.29, 1.28)	0.57 (0.29, 1.15)
Neither agree nor disagree	*Ref*	*Ref*	*Ref*	*Ref*	*Ref*	*Ref*
Agree	1.26 (0.66, 2.40)	1.02 (0.58, 1.79)	0.93 (0.59, 1.47)	1.33 (0.74, 2.38)	1.06 (0.62, 1.81)	1.01 (0.62, 1.64)
Strongly agree	1.48 (0.72, 3.04)	0.77 (0.40, 1.46)	0.73 (0.41, 1.31)	1.84 (0.85, 3.97)	1.72 (0.86, 3.44)	0.93 (0.50, 1.74)
**"Guns are the best way to protect yourself"**						
Strongly disagree	0.43 (0.13, 1.41)	0.92 (0.31, 2.79)	0.40* (0.17, 0.93)	1.64 (0.54, 4.98)	0.51 (0.16, 1.59)	0.62 (0.26, 1.47)
Disagree	1.41 (0.65, 3.09)	1.36 (0.67, 2.76)	0.93 (0.55, 1.56)	0.98 (0.48, 2.01)	1.43 (0.70, 2.92)	1.39 (0.79, 2.43)
Neither agree nor disagree	*Ref*	*Ref*	*Ref*	*Ref*	*Ref*	*Ref*
Agree	0.76 (0.42, 1.38)	1.03 (0.63, 1.69)	1.00 (0.66, 1.51)	1.14 (0.67, 1.96)	0.85 (0.52, 1.37)	1.29 (0.81, 2.05)
Strongly agree	0.65 (0.34, 1.23)	0.58 (0.31, 1.08)	0.88 (0.48, 1.60)	0.56 (0.28, 1.14)	0.43* (0.22, 0.84)	1.10 (0.60, 2.01)
**"No one should own AR-15 style semiautomatic rifles"**						
Strongly disagree	0.31** (0.14, 0.66)	0.42* (0.20, 0.85)	0.62 (0.33, 1.17)	0.46* (0.22, 0.95)	0.46* (0.24, 0.88)	0.38** (0.19, 0.78)

Disagree	0.54 (0.27, 1.05)	0.82 (0.45, 1.48)	0.91 (0.51, 1.60)	1.11 (0.58, 2.11)	0.66 (0.34, 1.30)	0.77 (0.43, 1.38)
Neither agree nor disagree	*Ref*	*Ref*	*Ref*	*Ref*	*Ref*	*Ref*
Agree	2.21* (1.06, 4.62)	2.63** (1.42, 4.87)	1.78* (1.04, 3.02)	2.10* (1.08, 4.09)	1.83* (1.01, 3.32)	1.59 (0.91, 2.76)
Strongly agree	7.25*** (2.96, 17.79)	5.73*** (3.04, 10.81)	5.44*** (3.09, 9.60)	2.73** (1.37, 5.43)	5.63*** (2.90, 10.90)	3.74*** (2.03, 6.89)
**"Gun control laws could prevent gun violence, like mass shootings, homicides, and suicides"**						
Strongly disagree	1.87 (0.85, 4.13)	1.20 (0.56, 2.57)	0.54 (0.27, 1.04)	1.95 (0.96, 3.95)	0.89 (0.44, 1.77)	1.69 (0.88, 3.23)
Disagree	1.98* (1.01, 3.86)	1.28 (0.70, 2.36)	0.86 (0.50, 1.49)	2.35* (1.19, 4.66)	1.43 (0.79, 2.57)	1.98* (1.12, 3.53)
Neither agree nor disagree	*Ref*	*Ref*	*Ref*	*Ref*	*Ref*	*Ref*
Agree	2.57** (1.25, 5.26)	2.71** (1.44, 5.09)	1.37 (0.81, 2.32)	2.09* (1.07, 4.10)	1.92* (1.10, 3.36)	2.52*** (1.52, 4.16)
Strongly agree	2.06 (0.69, 6.16)	4.35** (1.67, 11.33)	1.16 (0.56, 2.43)	2.67* (1.09, 6.53)	0.81 (0.36, 1.81)	3.08** (1.39, 6.80)
**"Gun control laws go against my Second Amendment rights"**						
Strongly disagree	1.73 (0.44, 6.84)	4.43 (0.95, 20.61)	1.11 (0.35, 3.48)	0.69 (0.19, 2.42)	1.58 (0.41, 6.01)	1.11 (0.41, 3.02)
Disagree	1.74 (0.55, 5.46)	1.45 (0.62, 3.38)	1.69 (0.89, 3.23)	1.08 (0.42, 2.79)	2.26 (0.97, 5.28)	1.32 (0.62, 2.83)
Neither agree nor disagree	*Ref*	*Ref*	*Ref*	*Ref*	*Ref*	*Ref*
Agree	0.34** (0.17, 0.67)	0.65 (0.34, 1.24)	0.94 (0.51, 1.74)	0.78 (0.38, 1.60)	0.89 (0.47, 1.69)	0.78 (0.43, 1.42)
Strongly agree	0.35* (0.12, 0.99)	1.10 (0.43, 2.83)	2.02 (0.81, 5.05)	2.01 (0.69, 5.88)	1.41 (0.56, 3.56)	0.39* (0.17, 0.90)
**"Gun control laws hurt law-abiding citizens"**						
Strongly disagree	1.71 (0.42, 7.06)	1.04 (0.25, 4.32)	2.46 (0.82, 7.36)	1.46 (0.45, 4.71)	1.45 (0.41, 5.17)	1.30 (0.46, 3.71)
Disagree	0.91 (0.33, 2.54)	0.98 (0.42, 2.27)	1.29 (0.65, 2.56)	1.08 (0.42, 2.73)	0.81 (0.34, 1.95)	1.89 (0.88, 4.06)
Neither agree nor disagree	*Ref*	*Ref*	*Ref*	*Ref*	*Ref*	*Ref*
Agree	1.24 (0.60, 2.59)	0.83 (0.42, 1.65)	0.98 (0.53, 1.82)	1.27 (0.59, 2.73)	0.89 (0.48, 1.66)	0.81 (0.45, 1.47)
Strongly agree	1.35 (0.42, 4.30)	0.50 (0.18, 1.42)	0.51 (0.21, 1.26)	0.38 (0.12, 1.20)	0.57 (0.23, 1.42)	0.98 (0.39, 2.50)

**"Gun laws would not work because guns are already in circulation"**						
Strongly disagree	0.98 (0.34, 2.85)	1.04 (0.27, 3.95)	1.26 (0.57, 2.80)	1.69 (0.59, 4.85)	3.76* (1.26, 11.22)	1.46 (0.59, 3.60)
Disagree	1.96 (0.96, 4.03)	1.36 (0.76, 2.46)	0.70 (0.43, 1.15)	1.40 (0.70, 2.81)	1.90 (0.95, 3.80)	1.13 (0.61, 2.08)
Neither agree nor disagree	*Ref*	*Ref*	*Ref*	*Ref*	*Ref*	*Ref*
Agree	1.84* (1.04, 3.24)	2.01** (1.20, 3.36)	1.25 (0.81, 1.95)	1.95* (1.16, 3.27)	1.33 (0.82, 2.17)	1.35 (0.84, 2.16)
Strongly agree	0.92 (0.45, 1.89)	1.92 (0.92, 4.01)	1.14 (0.57, 2.26)	1.15 (0.55, 2.42)	0.96 (0.47, 1.94)	1.11 (0.58, 2.14)
**Party Identity**						
Democrat	*Ref*	*Ref*	*Ref*	*Ref*	*Ref*	*Ref*
Independent	0.58 (0.30, 1.10)	0.76 (0.43, 1.34)	0.82 (0.48, 1.39)	0.49* (0.27, 0.90)	0.61 (0.34, 1.09)	0.66 (0.38, 1.14)
Republican	0.65 (0.35, 1.18)	0.79 (0.45, 1.37)	0.83 (0.51, 1.35)	1.05 (0.58, 1.89)	0.70 (0.41, 1.17)	0.77 (0.47, 1.28)
**Gun Ownership**						
Non-owner	*Ref*	*Ref*	*Ref*	*Ref*	*Ref*	*Ref*
Owner	0.99 (0.60, 1.62)	0.45*** (0.29, 0.70)	0.55*** (0.38, 0.79)	0.95 (0.55, 1.63)	0.66 (0.43, 1.03)	0.82 (0.54, 1.25)
**Gender**						
Cis-woman	*Ref*	*Ref*	*Ref*	*Ref*	*Ref*	*Ref*
Cis-man	0.59* (0.37, 0.94)	0.89 (0.59, 1.33)	0.92 (0.65, 1.30)	1.08 (0.68, 1.72)	0.67 (0.43, 1.03)	0.90 (0.60, 1.33)
Gender minority[g]	0.95 (0.16, 5.69)	0.37 (0.12, 1.10)	0.80 (0.15, 4.34)	0.13*** (0.04, 0.44)	1.98 (0.20, 19.55)	0.91 (0.14, 6.08)
**Race and Ethnicity**						
White, non-Hispanic	*Ref*	*Ref*	*Ref*	*Ref*	*Ref*	*Ref*
Black, non-Hispanic	0.37* (0.16, 0.85)	0.39** (0.19, 0.78)	0.52* (0.30, 0.92)	0.11*** (0.06, 0.21)	0.37*** (0.20, 0.68)	0.74 (0.36, 1.53)
Hispanic	0.71 (0.39, 1.27)	0.55* (0.32, 0.93)	1.41 (0.87, 2.30)	0.63 (0.36, 1.11)	0.84 (0.49, 1.44)	1.44 (0.89, 2.32)
Asian, non-Hispanic	0.62 (0.22, 1.73)	0.38 (0.12, 1.15)	1.18 (0.51, 2.72)	1.30 (0.38, 4.49)	0.75 (0.27, 2.09)	1.70 (0.53, 5.49)
Other or multiple	0.92 (0.35, 2.38)	1.04 (0.49, 2.19)	1.13 (0.56, 2.28)	0.26*** (0.12, 0.58)	0.71 (0.27, 1.86)	1.09 (0.49, 2.43)
**Age**						
18-29	*Ref*	*Ref*	*Ref*	*Ref*	*Ref*	*Ref*
30-44	0.91 (0.44, 1.85)	2.15* (1.15, 4.02)	0.94 (0.54, 1.65)	0.63 (0.35, 1.15)	0.57 (0.31, 1.06)	1.07 (0.61, 1.90)

45-59	1.06 (0.47, 2.38)	2.33* (1.17, 4.66)	0.81 (0.44, 1.49)	0.78 (0.38, 1.58)	0.77 (0.39, 1.51)	1.08 (0.58, 2.03)
60+	0.98 (0.40, 2.40)	3.98*** (1.79, 8.88)	0.81 (0.41, 1.58)	1.32 (0.56, 3.13)	1.16 (0.53, 2.54)	2.52* (1.21, 5.23)
**Education**						
High school or less	*Ref*	*Ref*	*Ref*	*Ref*	*Ref*	*Ref*
Some college	1.01 (0.62, 1.65)	1.22 (0.77, 1.91)	1.34 (0.89, 2.02)	0.92 (0.56, 1.51)	0.92 (0.59, 1.44)	0.69 (0.45, 1.05)
Bachelor's degree or higher	0.92 (0.51, 1.66)	0.76 (0.44, 1.31)	1.13 (0.72, 1.77)	1.55 (0.84, 2.89)	1.25 (0.73, 2.14)	1.05 (0.62, 1.77)
**Marital Status**						
Married	*Ref*	*Ref*	*Ref*	*Ref*	*Ref*	*Ref*
Never married	0.50* (0.28, 0.91)	0.87 (0.52, 1.46)	0.69 (0.43, 1.10)	0.54* (0.32, 0.92)	0.79 (0.47, 1.33)	0.68 (0.41, 1.13)
Other	0.96 (0.54, 1.72)	1.13 (0.67, 1.93)	1.06 (0.65, 1.71)	1.04 (0.53, 2.02)	0.95 (0.55, 1.66)	1.12 (0.68, 1.87)
**Income**						
Under $30,000	*Ref*	*Ref*	*Ref*	*Ref*	*Ref*	*Ref*
$30,000 to under $60,000	0.86 (0.48, 1.56)	1.19 (0.70, 2.00)	0.69 (0.42, 1.12)	1.08 (0.62, 1.89)	1.27 (0.73, 2.20)	0.93 (0.57, 1.52)
$60,000 to under $100,000	0.83 (0.40, 1.70)	1.63 (0.89, 2.97)	0.61 (0.37, 1.02)	0.70 (0.39, 1.27)	0.99 (0.54, 1.82)	0.82 (0.46, 1.47)
$100,000 or more	2.05* (1.01, 4.16)	1.49 (0.81, 2.75)	0.84 (0.49, 1.42)	1.06 (0.55, 2.05)	1.63 (0.87, 3.06)	1.00 (0.57, 1.78)
**Employment**						
Employed	*Ref*	*Ref*	*Ref*	*Ref*	*Ref*	*Ref*
Unemployed, looking for work	0.94 (0.34, 2.62)	1.96 (0.70, 5.50)	1.14 (0.57, 2.30)	1.21 (0.55, 2.66)	0.77 (0.31, 1.93)	1.67 (0.68, 4.10)
Retired	1.30 (0.63, 2.69)	1.13 (0.59, 2.15)	1.06 (0.62, 1.82)	1.59 (0.72, 3.52)	0.82 (0.41, 1.65)	0.40** (0.22, 0.74)
Other	0.75 (0.38, 1.47)	0.88 (0.52, 1.50)	0.91 (0.57, 1.48)	0.79 (0.44, 1.42)	0.77 (0.44, 1.35)	0.79 (0.47, 1.32)
**Region**						
Northeast	*Ref*	*Ref*	*Ref*	*Ref*	*Ref*	*Ref*
Midwest	0.70 (0.35, 1.40)	0.98 (0.54, 1.78)	0.93 (0.56, 1.55)	0.84 (0.44, 1.63)	0.72 (0.37, 1.42)	0.64 (0.35, 1.18)
South	0.72 (0.37, 1.43)	1.21 (0.67, 2.18)	1.81* (1.11, 2.96)	0.91 (0.47, 1.74)	1.08 (0.57, 2.03)	0.75 (0.43, 1.32)

West	0.72 (0.35, 1.50)	1.51 (0.78, 2.92)	1.43 (0.85, 2.43)	1.18 (0.61, 2.30)	1.11 (0.54, 2.29)	0.67 (0.37, 1.22)
**Metro Area Designation**						
Non-metro area	*Ref*	*Ref*	*Ref*	*Ref*	*Ref*	*Ref*
Metro Area	0.77 (0.43, 1.38)	0.71 (0.42, 1.20)	1.19 (0.75, 1.89)	0.81 (0.43, 1.52)	0.97 (0.59, 1.60)	1.03 (0.64, 1.65)

Note:*= p≤0.05,**= p≤0.01,***= p≤0.001

a = “Requiring universal background checks for all gun sales, including those at private sales, gun shows, and pawn shops.” b = “Requiring a waiting period of 3 to 5 days before someone can buy a gun.” c = “Raising the minimum age to buy any kind of gun from 18 to 21.” d = “Preventing people who have committed violent crimes from owning or using guns.” e = “Requiring a permit for concealed carry.” f = “Laws that allow law enforcement officers to temporarily remove guns from people that courts say are dangerous, like those at risk for suicide.” g = “Gender minority” represents respondents who self-identified as transmasculine, transfeminine, nonbinary, genderqueer, not exclusively female or male, or another gender.
